# Supplementary material for: Exploring the Relationship between the Engineering and Physical Sciences and the Health and Life Sciences by Advanced Bibliometric Methods
Source: PLoS One. 2014 Oct 31;9(10):e111530. doi: 10.1371/journal.pone.0111530 (PMC4216103; doi:10.1371/journal.pone.0111530)
Supplement: Table S1 — The 72 EPS research fields (WoS journal subject categories) used in the identification of research topics at the EPS-HLS interface. (DOCX) [file pone.0111530.s001.docx]

Table S1. The 72 EPS research fields (WoS journal subject categories) used in the identification of research topics at the EPS-HLS interface.

| acoustics  astronomy & astrophysics  automation & control systems  biophysics  chemistry, analytical  chemistry, applied  chemistry, inorganic & nuclear  chemistry, medicinal  chemistry, multidisciplinary  chemistry, organic  chemistry, physical  computer science, artificial intelligence  computer science, cybernetics  computer science, hardware & architecture  computer science, information systems  computer science, interdisciplinary applications  computer science, software engineering  computer science, theory & methods  construction & building technology  crystallography  electrochemistry  energy & fuels  engineering, aerospace  engineering, biomedical  engineering, chemical  engineering, civil  engineering, electrical & electronic  engineering, industrial  engineering, manufacturing  engineering, mechanical  engineering, multidisciplinary  engineering, petroleum  ergonomics  instruments & instrumentation  logic  materials science, biomaterials | materials science, ceramics  materials science, characterization & testing  materials science, coatings & films  materials science, composites  materials science, multidisciplinary  materials science, paper & wood  materials science, textiles  mathematical & computational biology  mathematics  mathematics, applied  mathematics, interdisciplinary applications  mechanics  metallurgy & metallurgical engineering  microscopy  mining & mineral processing  nanoscience & nanotechnology  nuclear science & technology  operations research & management science  optics  physics, applied  physics, atomic, molecular & chemical  physics, condensed matter  physics, fluids & plasmas  physics, mathematical  physics, multidisciplinary  physics, nuclear  physics, particles & fields  polymer science  robotics  social sciences, mathematical methods  spectroscopy  statistics & probability  telecommunications  thermodynamics  transportation  transportation science & technology |
| --- | --- |
