# Supplementary material for: Exploring the Relationship between the Engineering and Physical Sciences and the Health and Life Sciences by Advanced Bibliometric Methods
Source: PLoS One. 2014 Oct 31;9(10):e111530. doi: 10.1371/journal.pone.0111530 (PMC4216103; doi:10.1371/journal.pone.0111530)
Supplement: Table S2 — The 86 HLS research fields (WoS journal subject categories) used in the identification of research topics at the EPS-HLS interface. (DOCX) [file pone.0111530.s002.docx]

Table S2. The 86 HLS research fields (WoS journal subject categories) used in the identification of research topics at the EPS-HLS interface.

| agricultural engineering  agricultural experiment station reports  agriculture, dairy & animal science  agriculture, multidisciplinary  agronomy  allergy  anatomy & morphology  andrology  anesthesiology  audiology & speech-language pathology  behavioral sciences  biochemical research methods  biochemistry & molecular biology  biology  biophysics  biotechnology & applied microbiology  cardiac & cardiovascular systems  cell & tissue engineering  cell biology  clinical neurology  critical care medicine  dentistry/oral surgery & medicine  dermatology  developmental biology  emergency medicine  endocrinology & metabolism  entomology  evolutionary biology  fisheries  food science & technology  gastroenterology & hepatology  genetics & heredity  geriatrics & gerontology  gerontology  health care sciences & services  health policy & services  hematology  horticulture  immunology  infectious diseases  integrative & complementary medicine  marine & freshwater biology  mathematical & computational biology | medical informatics  medical laboratory technology  medicine, general & internal  medicine, research & experimental  microbiology  mycology  neuroimaging  neurosciences  nursing  nutrition & dietetics  obstetrics & gynecology  oncology  ophthalmology  ornithology  orthopedics  otorhinolaryngology  parasitology  pathology  pediatrics  peripheral vascular disease  pharmacology & pharmacy  physiology  plant sciences  primary health care  psychiatry  public, environmental & occupational health  radiology, nuclear medicine & medical imaging  rehabilitation  reproductive biology  respiratory system  rheumatology  social work  soil science  sport sciences  substance abuse  surgery  toxicology  transplantation  tropical medicine  urology & nephrology  veterinary sciences  virology  zoology |
| --- | --- |
